# Flipped and Peer-Assisted teaching: a new model in virtual anatomy education

**DOI:** 10.1186/s12909-024-05697-4

**Published:** 2024-07-03

**Authors:** Mohammad Afshar, Afagh Zarei, Mahdieh Rajabi Moghaddam, Hamed Shoorei

**Affiliations:** 1 Department of Anatomical Sciences, Faculty of Medicine, Birjand University of Medical Sciences, Birjand, Iran; 2 Medical Toxicology Research Center, Faculty of Medicine, Mashhad University of Medical Sciences, Mashhad, Iran; 3 Education Development Center, Birjand University of Medical Sciences, Birjand, Iran; 4 Pathology Department, Medical College, Birjand University of Medical Sciences, Birjand, Iran; 5 Cellular and Molecular Research Center, Birjand University of Medical Sciences, Birjand, Iran; 6 Clinical Research Development Unit of Tabriz Valiasr Hospital, Tabriz University of Medical Sciences, Tabriz, Iran

**Keywords:** Educational model, Active learning, Interactive tutorial, Flipped classroom, Peer-Assisted learning, Peer learning, Virtual learning, E-Learning

## Abstract

**Introduction:**

In response to the COVID-19 crisis, this study aimed to introduce a new virtual teaching model for anatomy education that combines Peer-Assisted Learning (PAL) and flipped classrooms, aligning with constructivist principles.

**Method:**

The Flipped Peer Assisted (FPA) method was implemented in a virtual neuroanatomy course for second-year medical students at Birjand University of Medical Sciences via a descriptive study. The method involved small groups of PAL, with peer learning serving as educational assistants and the teacher acting as a facilitator. Educational content was uploaded to the university’s learning management system (LMS). The opinion of medical students regarding the teaching method were evaluated using a 15-item questionnaire on a five-point Likert scale.

**Results:**

A total of 210 students participated in the instruction using the FPA method. The analysis of students’ scores revealed an average score of 26.75 ± 3.67 on the 30-point test. According to student feedback, this teaching method effectively motivated students to study, enhanced teamwork and communication skills, transformed their perspective on the anatomy course, provided opportunities for formative assessment and feedback, and demonstrated the teacher’s dedication to education.

**Conclusion:**

The FPA model demonstrates its effectiveness in transforming traditional classroom teaching and fostering teaching and learning in virtual environments, particularly during pandemics like COVID-19. This model holds promise for enhancing anatomy education in challenging circumstances.

## Introduction

Historically, anatomy has played a pivotal role in medical sciences education [1]. The teaching of fundamental anatomical knowledge is crucial for medical students and healthcare professionals alike [2]. Traditionally, the methods employed for teaching anatomy relied heavily on autopsies and lectures. However, advancements in educational technologies have paved the way for the integration of models, simulators, and the internet to enhance the anatomy learning experience [3]. Despite these advancements, there is still room for improvement in the field of anatomy education [4].

Furthermore, the global outbreak of Coronavirus Disease 2019 (COVID-19) has necessitated a shift from traditional to modern educational approaches [5]. Consequently, the provision of virtual anatomy education has become imperative [6]. Many active learning methods that were originally designed for face-to-face instruction have had to be adapted for virtual environments. While technologies like augmented reality, virtual reality, and 3D have shown potential in anatomy education [7, 8], their widespread application may be limited by various factors, including subject matter and contextual constraints [4].

In addition, the importance of teamwork, communication, and higher-order thinking skills has been emphasized in medical education, highlighting the limitations of traditional teaching methods in achieving these goals. As a result, there was a need to adopt educational approaches that would provide virtual anatomy education while enabling students to practice these skills and learn through effective feedback [9]. In this context, teachers play a crucial role. Good teachers expedite the learning process by offering relevant information, assessments, guidance, and examples. They motivate and guide learners, foster interactive discussions, and leverage their experience and skills to facilitate the learning process in a positive environment [10]. In the realm of electronic learning (e-learning), effective integration of existing technology-based methods becomes essential since there is no single, comprehensive e-learning model currently available [11–14].

Numerous educational techniques and approaches rooted in constructivism have been proposed, including flipped classrooms (FC) and peer-assisted learning (PAL). The term “flipped classroom” was first coined by American educators Jonathan Bergman and Aaron Sams in 2000 [15]. The FC Model presents a departure from the conventional teaching approach. Instead of the traditional method where teachers give lectures during class and assign homework, the FC Model delivers lectures outside of class and emphasizes in-class practice, which can be considered similar to homework. Another well-recognized framework, the FC, encompasses a set of teaching strategies that (1) move most of the direct instruction outside the classroom; (2) utilize classroom time for interactive and engaging learning activities; and (3) require students to participate in activities before and/or after class to fully leverage the benefits of classroom sessions [16]. This model involves inverting student activities with the support of new technologies, allowing students to engage more meaningfully with their teachers and enhancing face-to-face learning experiences [17]. Indeed, the FC, which holds great potential in e-learning for healthcare, is supported by active learning theory and promotes critical thinking, problem-solving, and experimentation [18]. Furthermore, the FC aligns with the Theory of Educational Differentiation, which emphasizes personalized teaching to cater to the diverse needs of learners. Differentiated instruction involves adapting teaching methods based on students’ needs, interests, and abilities to enhance their learning and academic achievement [19].

Despite its numerous advantages, the FC also faces certain challenges. One significant hurdle is ensuring that all students arrive prepared for class, having actively engaged with the pre-class material. Without this preparation, the planned active learning strategies for class time may not yield the desired results. Additionally, some students may perceive the FC as an unfair or unreasonable approach. On the other hand, teachers may find FCs demanding due to the time-consuming nature of the process, difficulties in task planning and management, and the associated increase in workload [20]. As a potential solution to these issues, we hypothesize that peer-assisted learning could be a suitable approach.

PAL, in contrast, is defined as the process of actively assisting and supporting one another among peers of equal status or matched companions. It involves individuals from similar social groups who are not professional educators, helping each other learn and, in turn, enhancing their own learning [21]. Therefore, PAL is an approach in which students provide assistance to their peers in the learning process. It encompasses different forms, including peer tutoring, peer consulting, peer education, peer modeling, peer assessment, and peer monitoring [21].

Against this backdrop, in this study, we present a novel virtual teaching model that combines PAL and FCs. This model was implemented and evaluated during the crisis era.

## Materials and methods

### Participants and study design

This descriptive study aimed to examine the impacts of the FPA (Flipped Peer-Assisted) method, a novel educational model, on the neuroanatomy course at Birjand University of Medical Sciences (BUMS) over three consecutive semesters from 2020 to 2021.

In this study, a total of 210 fourth-semester medical students (female students: *n* = 120, with an average age of 20.7 years, and male students: *n* = 90, with an average age of 20.9 years) were selected as participants. The inclusion criteria for this study were individuals who had successfully completed the third semester and entered the fourth semester of the medical program, as well as those who had not previously failed the neuroanatomy course. The FPA method was implemented, drawing inspiration from the flipped classroom approach, where small groups of students engaged in peer-assisted learning. Additionally, the model incorporated feedback sessions to foster reflection and learning, with the teacher assuming the role of a facilitator as needed. The implementation of the FPA method consisted of three steps. In the first step, instructional design was carried out, and electronic content was produced. The second step involved the actual implementation, which commenced with an online orientation class. Students were divided into groups of 10, and within each group, one student was chosen as the peer learning based on their educational level and communication skills. Given that each semester had 70 students, a total of 10 groups and seven peer learning were selected. The mean scores of students in different groups were equated. The educational content for each lesson was uploaded to the university’s learning management system (LMS) for one week. Three days after the content was uploaded, an online meeting was held between the teacher and the pre-teachers to ensure their understanding of the material and emphasize key points. Two days later, the remaining students who had studied the uploaded content had an online class with their respective peer learning, which focused on questions and answers and highlighting important concepts. Finally, an online session was conducted with all the groups, evaluating students’ learning through questions and providing additional explanations if necessary. The peer learning’s ‘s performance in delivering the educational content and interacting with students was also evaluated, and feedback was provided to improve their performance. This process was repeated every week, and students received feedback from both the professor.

### Evaluation of the study

To collect students’ opinions about the teaching method, a 15-item questionnaire was administered. The questionnaire utilized a five-point Likert scale, ranging from “very high” to “very low.” The content validity of the questionnaire was established through the confirmation of ten medical educationists, and its reliability was assessed using Cronbach’s alpha, which yielded acceptable results. Additionally, an open-ended question was included in the LMS to gather students’ overall feedback and opinions regarding their learning experience with this teaching model.

### Instruments

At the end of the semester, students’ learning was assessed using a 30 multiple choice question test designed according to the test blueprint which was a representative test of all course objectives and was approved in the anatomy department. These tests were different from each other in each semester. However, it was designed according to the blueprint of the exam, and by checking it in the anatomy department, it was ensured that the educational goals of the course were covered.

## Results

The results of the study indicate several important findings regarding the implementation of the educational model. A total of 210 medical students experienced instruction through this method over three consecutive semesters. Table 1 presents the students’ opinions about the teaching method.

**Table 1:** The results of students’ opinions about the FPA model

	Questions	Very High	High	Medium	Low	Very Low
1	To what extent does this teaching model agree with the learners’ expectations of instruction?	55%	41%	4%	-	-
2	To what extent does this teaching model interest and motivate learners?	45%	41%	14%	-	-
3	To what extent does the teacher successfully convey concepts to learners through this teaching model?	39%	54%	7%	-	-
4	To what extent does the teacher successfully convey concepts to learners through this teaching model?	48%	41%	11%	-	-
5	To what extent does the teacher succeed in building knowledge in the learner’s mind through this teaching model?	36%	54%	10%	-	-
6	How effective is this model in learning compared to other virtual teaching methods?	50%	46%	2%	2%	-
7	How is this model effective in learning compared to face-to-face teaching methods?	30%	46%	18%	4%	2%
8	How much does the feedback received through this teaching method affect students’ learning?	38%	54%	8%	-	-
9	To what extent does this teaching method make learners use thinking and analysis skills?	29%	46%	21%	4%	-
10	How much did this tutorial help learners quickly retrieve information from their minds?	34%	52%	11%	3%	-
11	To what extent can this teaching method improve learners’ teamwork skills?	30%	46%	13%	5%	5%
12	To what extent can this teaching method improve learners’ communication skills?	34%	41%	18%	5%	2%
13	How satisfied may learners be with this teaching model?	48%	45%	7%	-	-
14	To what extent do learners like to use this model in other situations?	39%	45%	9%	2%	5%
15	To what extent can this learning model be a substitute for a face-to-face classroom?	39%	29%	23%	5%	4%

### Five categories were identified based on the students’ feedback

#### Study techniques

Students mentioned that this method compelled them to utilize effective study techniques such as repetition, review, and peer review. They also emphasized the need for accurate and timely studying.

#### Teamwork and communication skills

Students reported an increase in group companionship and cooperation, as well as a sense of responsibility. They mentioned that this method allowed for task assignment among students and improved their verbal communication skills. Additionally, all students made efforts to promote the group’s success.

#### Transformation

According to the students, this teaching method successfully changed their perspective towards the anatomy course. They expressed gratitude for shifting their negative opinions to a more positive outlook, particularly regarding the fetal anatomy component.

#### Formative assessment and feedback

Students appreciated the opportunities provided by this teaching model for peer questioning and answering, evaluation by peers, interaction with the teacher through questioning and answering, and problem-solving to achieve a deep understanding of the material. They also mentioned that this method alleviated concerns about the final exam. Group evaluation was implemented, fostering a sense of competition among students.

#### Teacher role

Although the educational model was designed to be student-centered, students still recognized and appreciated the role of the teacher in its implementation. They considered the adoption of this method as a sign of respect for the student, viewing the teacher as a role model who is duty-oriented, concerned, and attentive to education. The analysis of students’ scores on the 30-point test revealed an average score of 26.75 ± 3.67.

## Discussion

The COVID-19 pandemic has necessitated a shift to online learning and assessment methods. Distance learning content focusing on active learning has been a significant concern for teachers. It is crucial for educators to transition from a traditional approach of acquiring knowledge to one that emphasizes knowledge construction. In response to the COVID-19 outbreak, various studies have indicated a widespread move towards virtual education to support student learning [6, 11].

The flipped classroom has gained traction as a pedagogical approach in higher education, with several studies highlighting positive student outcomes [22, 23].

The flipped classroom model transforms anatomy teaching by replacing large-scale lectures with interactive, team-based classes. The incorporation of multimodal digital resources and multimedia group assignments in the flipped classroom model fosters digital literacy and enables students to engage in authentic learning experiences, contributing to pedagogical innovation [24]. So, the FPA model as an active learning approach helps learners dedicate part of their time to receiving feedback, engaging in reflection, and taking action based on that feedback. The primary purpose of feedback in this model is to provide an opportunity for setting learning goals and reinforcing learning [15, 17, 21, 25, 26]. A systematic review conducted by Kazeminia et al. in 2022 highlighted the positive impact of the FC method on teaching anatomy; the review revealed numerous advantages associated with this approach, including increased satisfaction, academic achievement, self-confidence, and overall outcomes in anatomy curricular [27]. A study conducted in China demonstrated an increase in the adoption of active learning methods, such as FCs, individualized tutoring, and small group discussions, during the COVID-19 pandemic [28]. Interactions in these active learning sessions primarily took place through real-time voice, text, or teaching management platforms like Rain Classroom, Xuexitong, or Blackboard. In the context of English classroom teaching, a study introduced the SPOC-based (Small Private Online Course) FC model as an effective learning approach [29]. Consistent with our findings, one research revealed that the students exhibited a notable level of enthusiasm towards the pre-class activities, which effectively fostered their active participation and attentiveness during the in-class sessions [30].

On the one hand, studies conducted in India comparing e-learning methods during the COVID-19 crisis have found that learners generally express agreement with online discussions facilitated through virtual classrooms [6]. From the learners’ perspective, e-learning is seen as a complement to traditional education, forming integrated educational methods [31]. Constructive alignment of integrated methods in an e-learning model can contribute to effective educational design. Research has shown that learners’ satisfaction is influenced by factors such as teachers’ attitudes towards e-learning, course flexibility, course quality, course utility, perceived ease of use, and variety of assessments [32]. According to a study carried out at The College of Health-Care Professions in Bolzano, Italy, it was suggested that incorporating online learning activities can enhance the effectiveness of didactic lectures. This approach not only requires teachers to create well-structured courses but also encourages early student engagement with the course material, thereby preventing a passive role during in-class sessions [33]. Furthermore, in a study conducted by Telford and Senior in 2017, healthcare students who experienced a combination of e-learning and flipped classroom instructional approaches reported positive outcomes and a favorable learning experience [18].

Advancements in concurrent distance learning and shared technologies, such as blogs, bulletin boards, chats, emails, and distance conferencing, have made it easier to access shared learning experiences. Quantitative and qualitative studies in collaborative medical education have demonstrated higher levels of learner satisfaction, knowledge progression, self-awareness, understanding of concepts, achievement of course goals, and changes in practice.

It is important to recognize that in “e-learning,” the e-learner plays a central role. The use of information and communication technology by the new generation of digital natives distinguishes them from previous generations of learners and teachers. Therefore, effective e-medicine learning requires a balance between real-world practice and providing adequate learning opportunities. However, it is crucial to note that many challenges related to the use of technology in medical education are not solely attributable to the technology itself but to the educational approaches employed.

A significant limitation of this study was the absence of a control group for comparing the effects of the FPA model with other models. However, the application of this model over three consecutive semesters and the high satisfaction rate among students were notable strengths. Future high-quality studies are recommended to compare the effects of this method with other approaches. Additionally, exploring the applicability of this model in other fields and subjects is suggested.

## Conclusion

The FPA model is a student-centered approach that encourages students to employ diverse learning techniques. It recognizes the important mentoring role of teachers in the learning process. The FPA model demonstrates remarkable effectiveness in deconstructing traditional classroom teaching and reconstructing teaching and learning methods through the profound integration of classroom instruction and modern educational technologies. This model is particularly valuable during times of crisis, such as the ongoing pandemic.

## Data Availability

All data generated and/or analyzed during the current study are available from the corresponding authors on responsible request.
